# Application of radiomics and machine learning to thyroid diseases in nuclear medicine: a systematic review

**DOI:** 10.1007/s11154-023-09822-4

**Published:** 2023-07-12

**Authors:** Francesco Dondi, Roberto Gatta, Giorgio Treglia, Arnoldo Piccardo, Domenico Albano, Luca Camoni, Elisa Gatta, Maria Cavadini, Carlo Cappelli, Francesco Bertagna

**Affiliations:** 1 Nuclear Medicine, ASST Spedali Civili di Brescia, P.le Spedali Civili, 1, Brescia, 25123 Italy; 2 Dipartimento di Scienze Cliniche e Sperimentali, Università degli Studi di Brescia, Brescia, Italy; 3 Clinic of Nuclear Medicine, Imaging Institute of Southern Switzerland, Ente Ospedaliero Cantonale, Bellinzona, Switzerland; 4 Department of Nuclear Medicine and Molecular Imaging, Lausanne University Hospital, University of Lausanne, Lausanne, Switzerland; 5 Faculty of Biomedical Sciences, Università della Svizzera italiana, Lugano, Switzerland; 6 Department of Nuclear Medicine, Ospedali Galliera, Genoa, Italy; 7 Nuclear Medicine, ASST Spedali Civili di Brescia and Università degli Studi di Brescia, Brescia, Italy; 8 Unit of Endocrinology and Metabolism, ASST Spedali Civili di Brescia and Università degli Studi di Brescia, Brescia, Italy

**Keywords:** Thyroid, Machine learning, Radiomics, Texture analysis, Positron emission tomography

## Abstract

**Background:** In the last years growing evidences on the role of radiomics and machine learning (ML) applied to different nuclear medicine imaging modalities for the assessment of thyroid diseases are starting to emerge. The aim of this systematic review was therefore to analyze the diagnostic performances of these technologies in this setting. **Methods:** A wide literature search of the PubMed/MEDLINE, Scopus and Web of Science databases was made in order to find relevant published articles about the role of radiomics or ML on nuclear medicine imaging for the evaluation of different thyroid diseases. **Results:** Seventeen studies were included in the systematic review. Radiomics and ML were applied for assessment of thyroid incidentalomas at ^18^ F-FDG PET, evaluation of cytologically indeterminate thyroid nodules, assessment of thyroid cancer and classification of thyroid diseases using nuclear medicine techniques. **Conclusion:** Despite some intrinsic limitations of radiomics and ML may have affect the results of this review, these technologies seem to have a promising role in the assessment of thyroid diseases. Validation of preliminary findings in multicentric studies is needed to translate radiomics and ML approaches in the clinical setting.

## Introduction

Thyroid diseases are a heterogeneous group of conditions which includes both benign and malignant diseases, with an increasing in the incidence of both of them in the recent years. Notably, differentiated thyroid cancer (DTC) is the most common endocrine malignant neoplasm worldwide, while Hashimoto’s thyroiditis is the most common autoimmune form of thyroid disease [1].

The diagnosis of thyroid pathologies is based primarily on the assessment of its function with laboratory blood test, in particular for thyroiditis or hyperthyroidism [2]. Moreover, the anatomical evaluation of the organ with the well-established ultrasonography (US) is mandatory to assess the presence of nodules, possible expression of thyroid cancer, or other pathological conditions and therefore this imaging modality has experienced an increasing relevance in the last decades [3].

In this scenario, the role of nuclear medicine for the diagnosis and treatment of thyroid conditions is central. Thyroid scintigraphy with ^99m^Tc-pertechnetate enables the functional evaluation of the gland, allowing the differentiation between thyroiditis and hyperthyroidism, but also detecting the presence of ectopic thyroid tissue [4]. Moreover, ^131^I is mandatory for the management of DTC, since its pivotal role for the therapy but also the stage or the restage of the disease [5–9]. Beside single photon imaging, positron emission tomography (PET) has continuously spread its indication for the evaluation of DTC and associated conditions, and in the recent years many different tracers have been proposed for the assessment of such diseases [7–12].

Recently, an increase in the extraction of specific quantitative features from PET and scintigraphic images, called radiomics or texture analysis, is being experienced and researches in this field are focusing on its diagnostic and prognostic role in a wide range of pathological conditions, and the thyroid does not make any exception [13–14]. Similarly, machine learning (ML) is a hot topic of recent clinical research and focuses on the development of algorithms that can use different combinations of features in order to predict a specific target [15–16].

The aim of this systematic review is therefore to evaluate the role of radiomics and ML for the assessment of thyroid diseases.

## Materials and methods

### Search strategy

A wide literature search of the PubMed/MEDLINE, Scopus and Web of Science databases was made in order to find significant published articles concerning the role of radiomics and ML for the assessment of thyroid diseases. The algorithm used for the research was the following: (“thyroid”) AND (“radiomics” OR “texture” OR “textural” OR “machine learning”).

No beginning date limit was applied to the search, and it was updated until 01 February 2023. Only articles in the English language were considered and preclinical studies, conference proceedings, reviews or editorials were excluded. To expand our search, the references of the retrieved articles were also screened for additional papers.

### Study selection

Two researchers (F.D. and R.G.) independently reviewed the titles and abstracts of the retrieved articles. The same two researchers then independently reviewed the full-text version of the remaining articles to determine their eligibility for the inclusion.

### Quality assessment

The quality assessment of these studies, including the risk of bias and applicability concerns, was carried out using Quality Assessment of Diagnostic Accuracy Studies version 2 (QUADAS-2) evaluation [17].

### Data extraction

For each included study, data concerning the basic study (author names, year of publication, country of origin, design of the study, radiotracer used and number of patients), the type of scan used and its setting were collected. The main findings of the articles included in this review are reported in the Results section.

## Results

### Literature search

**Fig. 1:**
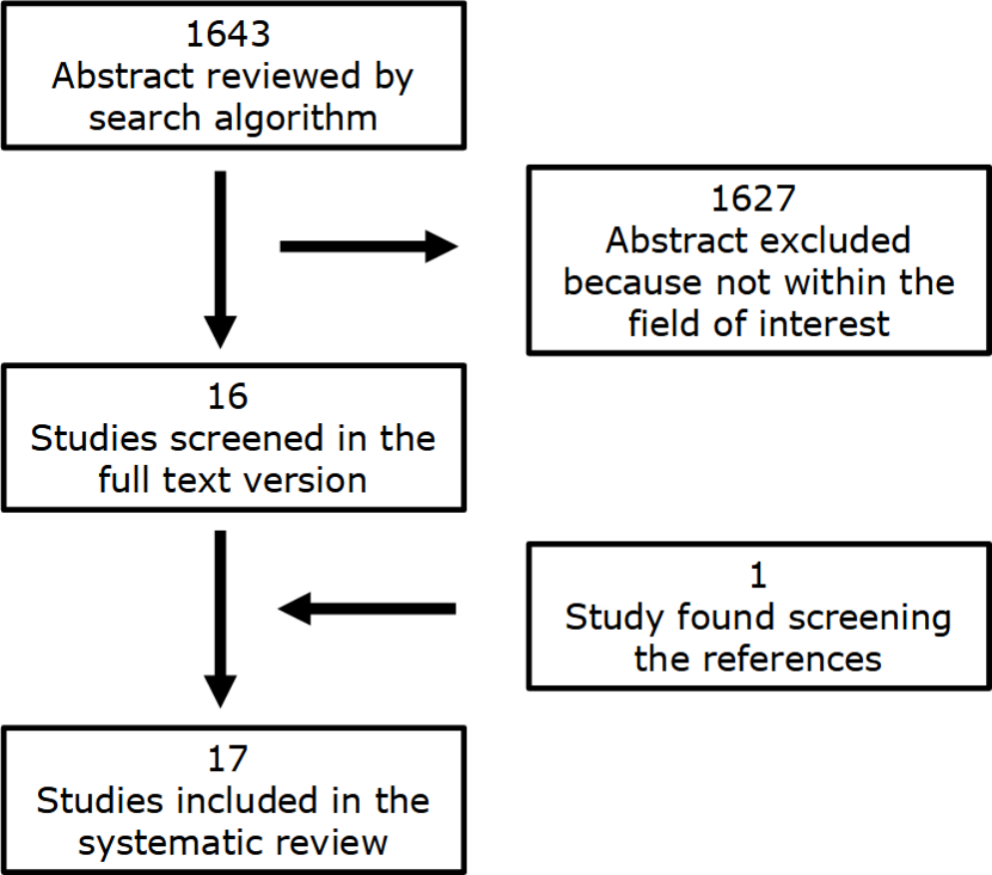
Flowchart of the research of eligible studies on the role of radiomics or ML for the assessment of pathological conditions of thyroid

A total of 1643 articles were extrapolated with the computer literature search and, by reviewing the titles and abstracts, 1627 of them were excluded because the reported data were not within the field of interest of this review. Sixteen articles were therefore selected and retrieved in full-text version [18–33] and one additional study was also found screening the references of these articles (Fig. 1) [34]; as a consequence, the total number of studies evaluated in the review was 17.

**Fig. 2:**
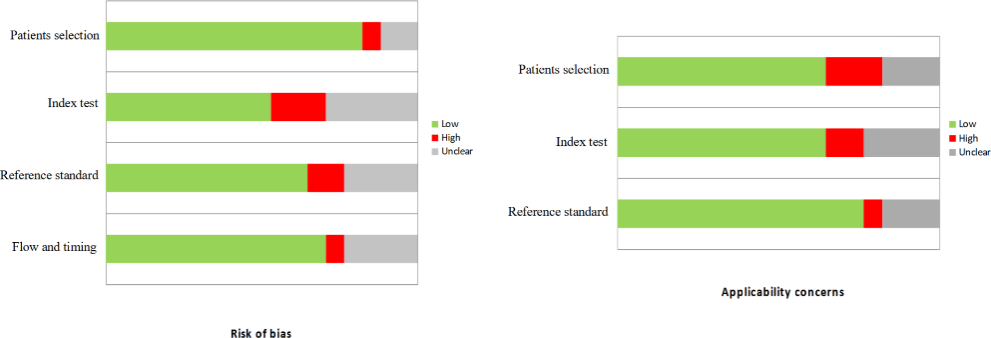
QUADAS-2 quality assessment for risk of bias and applicability concerns for the studies considered in the review

In general, the quality assessment using QUADAS-2 evaluation underlined the presence of unclear risk of bias and applicability concerns in some of the studies for what concerns patients selection, index test, reference standard and flow and timing. Nevertheless, only a small amount of studies were characterized by the presence of high risks of bias or applicability (Fig. 2).

Among the total number of studies included in the systematic review, 14 were of retrospective nature [18–24, 26–28, 30, 32–34], whereas one had a prospective design [25] and in two cases it was not specified the nature of the study [29, 31]. Ten studies focused on PET imaging [18–26, 34], 8 with PET/computed tomography (PET/CT) hybrid tomographs [19–20, 22–26, 34] and 2 with both PET/CT and PET [18, 21]. Furthermore, 7 studies focused on single photon imaging [27–33] and in particular 3 were performed with single photon emission computed tomography (SPECT) [27, 29, 31] while 4 were performed with planar scintigraphic scans [28, 30, 32–33].

Speaking about radiopharmaceuticals, 9 of the studies that focused on PET imaging were performed with ^18^ F-fluorodeoxyglucose (^18^ F-FDG) [19–26, 34], while in 1 case both ^68^Ga-DOTATOC or ^68^Ga-DOTATATE were used [18]. Moreover, in the case of scintigraphic imaging, ^131^I was used in 2 studies [28–31] while in 4 other it was reported the use of ^99m^Tc-pertechnetate [29–[30, 32]–33]; lastly, in 1 case the radiotracer was not specified [27].

The main characteristics of the studies and their results are briefly presented in Tables 1 and 2.

**Table 1:** Characteristics of the studies considered for the review

First author	Ref. N.	Year	Country	Study design	Radiopharmaceuticals	N. Pts.	Sex M:F
**PET studies**							
Kim SJ	34	2015	Korea	Retrospective	^18^ F-FDG	200	43:157
Lapa C	18	2015	Germany	Retrospective	^68^Ga-DOTATOC ^68^Ga-DOTATATE	12	9:3
Sollini M	19	2017	Italy	Retrospective	^18^ F-FDG	55	20:35
Nakajo M	20	2018	Japan, Germany	Retrospective	^18^ F-FDG	114	33:81
Werner RA	21	2018	Germany, USA, Japan, Hungary	Retrospective	^18^ F-FDG	18	12:6
Aksu A	22	2020	Turkey	Retrospective	^18^ F-FDG	60	ns
Ceriani L	23	2020	Switzerland, Italy	Retrospective	^18^ F-FDG	104	37:67
Giovanella L	24	2021	Switzerland, Italy	Retrospective	^18^ F-FDG	78	20:58
De Koster EJ	25	2022	The Netherlands, Italy	Prospective	^18^ F-FDG	123	21:102
Dondi F	26	2022	Italy	Retrospective	^18^ F-FDG	221	72:149
**Other studies**							
Ma L	27	2019	China	Retrospective	ns	2888	ns
Kavitha M	28	2020	Japan, South Korea	Retrospective	^131^I	230	ns
Liu Y	29	2020	China	ns	^99m^Tc-pertechnetate	136	ns
Currie G	30	2021	Australia, USA, Pakistan	Retrospective	^99m^Tc-pertechnetate	123	12:111
Guo Y	31	2021	China	ns	^131^I	446	87:359
Qiao T	32	2021	China	Retrospective	^99m^Tc-pertechnetate	1430	ns
Yang P	33	2021	China	Retrospective	^99m^Tc-pertechnetate	3389	168:3221

N.: number; Pts: patients; Ref: reference; M: male; F: female; ns: not specified

**Table 2:** Results and main findings of the studies considered for the review

First author	Type of scan	Number of scanners	Setting	Patients characteristics	Performance validation methods	ML/statistical techniques	Features with best performances	Main findings
**PET studies**								
Kim SJ	PET/CT	1	Evaluation of citologically indeterminate thyroid nodules	151 without malignancy and 49 with malignancy	Training set	Kruskall-Wallis	HF	Intratumoral heterogeneity could characterize thyroid nodules with inconclusive FNAB.
Lapa C	PET and PET/CT	3	Evaluation of iodine refractory DTC or MTC treated with PRRT	4 MTC and 8 iodine refractory DTC	Training set	Survival models	Contrast, Entropy, Grey Level Non Uniformity, High Grey Level Zone Emphasis, Intensity Variation, Short Run Emphasis, Short Run High Level Grey Emphasis, Short Zone High Grey Level Emphasis, Short Zone Low Grey Level Emphasis	Tumor heterogeneity could be a predictor of response to PRRT.
Sollini M	PET/CT	2	Assessment of thyroid incidentalomas at PET/CT	32 without malignancy and 18 with malignancy	Training set	Fisher test, ANOVA	Skewness, Kurtosis, Correlation_GLCM_	Texture analysis seems to be able to stratify thyroid incidentalomas with respect to the risk of malignancy.
Nakajo M	PET/CT	1	Prediction of the risk of recurrence of DTC	88 ^18^ F-FDG avid tumors and 26 non-avid	Training set	Mann Whitney, Chi square	IV, SVZ, ZP	When MTV is higher than 10, the combined use of SUV-related, volumetric and texture parameters increases the identification of patients with high risk of recurrence.
Werner RA	PET and PET/CT	3	Prognostic evaluation in patients with MTC treated with TKI	1 with hereditary MTC and 17 without	Training set	Survival models	Complexity, Contrast	Baseline complexity and TLG are independent prognosticators for OS.
Aksu A	PET/CT	1	Assessment of thyroid incidentalomas at PET/CT	32 without malignancy and 28 with malignancy	Cross-fold validation	RF, KNN, naive bayes, DT and support vector machine	GLRLM_RLNU_	Texture analysis may be more useful than SUVmax in predicting the malignancy of thyroid incidentalomas.
Ceriani L	PET/CT	2 of the same model	Assessment of thyroid incidentalomas at PET/CT	77 without malignancy and 30 with malignancy	Cross-fold validation	Logistic regression	Shape_Sphericity	The proposed multiparametric radiomics model showed good performance in stratifying the risk of malignancy of incidentalomas.
Giovanella L	PET/CT	2	Evaluation of citologically indeterminate thyroid nodules	55 without malignancy and 23 with malignancy	Cross-fold validation	LASSO logistic regression	Shape_Sphericity, GLCM_Autocorrelation	The proposed multiparametric model increased the accuracy of risk stratification compared to Bethesda system and PET/CT alone.
De Koster EJ	PET/CT	20	Evaluation of citologically indeterminate thyroid nodules	99 without malignancy and 24 with malignancy	Cross-fold validation	Elastic net regression	/	Radiomic analysis did not contribute to the additional differentiation of thyroid nodules.
Dondi F	PET/CT	2	Assessment of thyroid incidentalomas at PET/CT	150 without malignancy and 71 with malignancy	Cross-fold validation	Logistic regression	GLCM-related features	Some radiomics features were able to predict with certain good accuracy the final diagnosis of incidentalomas. A good overlap in the extraction of these features between two different scanner was reported.
**Other studies**								
Ma L	SPECT	1	Classification of thyroid diseases at scintigraphy	780 with Grave’s disease, 438 with Hashimoto disease, 810 with subacute thyroidits and 860 normal	Independent internal testing set	8 different ANNs	/	The proposed network is efficient for the diagnosis of thyroid diseases with SPECT images.
Kavitha M	Planar whole-body	1	Recognition of metastatic lymph nodes in DTC	2481 remnant tissue regions and 500 metastatic lymph nodes regions	Cross-fold validation	ANN	/	The proposed model offers excellent diagnostic performances for the assessment of metastatic lymph nodes.
Liu Y	SPECT	1	Classification of thyroid diseases at scintigraphy	65 with hyperthyroidism, 43 with hypothyroidism and 28 normal	Independent internal testing set	4 different ANNs	/	The four deep learning models are efficient for the classification of thyroid diseases with SPECT images.
Currie G	Planar	ns	Classification of thyroid diseases at scintigraphy	9 with hypothyroidism, 22 with Grave’s disease, 9 with multinodular goitres, 2 with nodular thyroids, 3 with goitres, 11 with reduced or absent uptake, 7 with autonomous glands with contralateral suppression, 24 with cold nodules, 8 with hot nodules and 28 normal	Independent internal testing set	ML and deep learning ANNs	/	The proposed ML algorithm can improve accuracy as second readers system. DL algorithms can be developed to improve accuracy in the absence of biochemistry results.
Guo Y	SPECT	1	Classify and diagnose the residual thyroid tissue after thyroidectomy	346 with residual tissue and 100 without residual tissue	Independent internal testing set	ANN	/	The proposed method has good performances for the assessment of residual thyroid tissue.
Qiao T	Planar	1	Classification of thyroid diseases at scintigraphy	175 with no thyroid disease, 834 with Grave’s disease and 421 with subacute thyroiditis	Independent internal testing set	3 different ANNs	/	Deep learning models perform well in the diagnosis of Grave’s disease and subacute thyroiditis.
Yang P	Planar	2 of the same model	Classification of thyroid diseases at scintigraphy	1420 diffusely increased uptake, 1177 diffusely decreased uptake, 135 focal increased uptake and 657 heterogeneous uptake	Independent internal and external testing set	4 different ANNs	/	The proposed AI model has good accuracy in the classification of thyroid disease .

N.: number; FNAB: fine needle aspiration biopsy; DTC: differentiated thyroid cancer; MTC: medullary thyroid cancer; PRRT: peptide receptor radionuclide therapy; MTV: metabolic tumor volume; OS: overall survival; SUV: standardized uptake value; AI: artificial intelligence; ML: machine learning; RF: random forest; KNN: k nearest neighbor; DT: decision tree; LASSO: least absolute shrinkage and selection operator; ^18^ F-FDG: ^18^ F-fluorodeoxyglucose; ANN: artificial neural network; HF: heterogeneity factor; GLCM: grey level co-occurrence matrix; IV: intensity variability; SVZ: site-zone variability; ZP: zone%; GLRLM_RLNU_: gray-level run-length matrix run length non-uniformity

### PET/CT studies

As mentioned, several studies evaluated different thyroid conditions by applying radiomics and ML to positron imaging [18–26, 34], demonstrating the possible role of such techniques in these areas of research. A list and a legend of the radomics features with best performances are presented in Table 3.

**Table 3:** Legend of the radomics features with best performances

Feature’s full name	Feature’s abbreviation used in the original work	Feature’s meaning
Heterogeneity factor	HF	Variation in glucose metabolism over the entire volume consiedered
Contrast	/	Measures the difference of the grey value when going to the next voxel
Entropy	/	A measure for the grade of derangement
Grey Level Non Uniformity	/	Measures the similarity of grey level values
High Grey Level Zone Emphasis	/	Measures the distribution of high grey level values
Intensity Variation	/	Describes the variation of the intensity of different substructures (zones)
Short Run Emphasis	/	Describes the distribution of runs
Short Run High Level Grey Emphasis	/	Measures the joint distribution of small runs and low grey level values
Short Zone High Grey Level Emphasis	/	Measures the joint distribution of short zones and high grey level values
Short Zone Low Grey Level Emphasis	/	Measures the joint distribution of small zones and low grey level values
Skewness	/	Represents the symmetry of distribution of grey levels
Kurtosis	/	Is a measure of peakedness in the grey level distribution
Correlation grey level co-occurrence – related	GLCM – related, Correlation_GLCM_, GLCM_Autocorrelation	The GLCM is a matrix that expresses how combinations of discretised grey levels of neighbouring pixels are distributed along one of the image directions
Intensity variability	IV	Measures the similarity in pixel intensities throughout the image
Size-zone variability	SVZ	Measures the similarity in zone sizes
Zone%	ZP	Measures the homogeneity
Complexity	/	Refers to the visual information content of a texture; a texture is considered complex if the information content is high
Contrast	/	Measures the intensity difference between neighboring regions
Gray-level run-length matrix run length non-uniformity	GLRLM_RLNU_	Assesses the distribution of runs over the run lengths
Shape_Sphericity		Depicts the geometric properties of the lesion in terms of sphericity

#### Assessment of thyroid incidentalomas

One of the most explored field of application of radiomics in thyroid PET/CT imaging is the assessment of ^18^ F-FDG avid thyroid incidentalomas (TI). In this setting, the first research on this topic was proposed by *Sollini et al.* [19], reporting that some specific radiomics feature (RF) related to “Compacity” were significantly different between TIR categories and that “Skewness” was different between benign and malignant nodules. Moreover 3 RF (“Skewness”, “Kurtosis”, “Correlation_GLCM_”) were selected, in addition to standardized uptake value (SUV)-related and volumetric parameters, as potential predictors with high sensitivity. In the same setting *Aksu et al.* [22] revealed that 21 RF were significantly different between malignant and benign nodules and “GLRLM_RLNU_” was reported as the feature with the best discriminating power with high specificity, positive predictive value (PPV) and negative predictive value (NPV). Moreover, the authors proposed a random forest model including this feature and SUVmax with good performances in the classification of TI (area under the curve [AUC] 0.849). More recently, *Ceriani et al.* [23] performed a similar analysis, including however the evaluation of the influence of different scanner on the extraction of RF. In this setting, only 54/107 RF were statistically reproducible between the two PET/CT scanner included in the study and “Shape_Sphericity” was reported as an affordable classificator. Furthermore, a predictive model with total lesion glycolysis (TLG), SUVmax and “Shape_Sphericity” was built by the authors. Similarly, *Dondi et al.* [26] evaluated the influence of different scanner on the extraction of RF and their ability to predict the final diagnosis of TI. In this setting, they reported that 9/42 RF had apparent correlation with the scanner used for their extraction, with cross-correlation maps that were quite similar between the two scanners. After bivariate analysis performed for single scanners and considering both of them together, none of the RF obtained an optimal AUC above 0.8 and, in general, higher AUCs value were visible on a particular scanner. Generally speaking, GLCM-related features were the ones with best perfomances.

#### Evaluation of citologically indeterminate thyroid nodules

Fine-needle aspiration biopsy is an accurate and essential method for the assessment of thyroid nodules, however in about 30% of the cases its results remain inconclusive or indeterminate. In this setting, *Kim et al.* [34] were the first to propose a paper to evaluate the predictive role of distributive ^18^ F-FDG heterogeneity to characterize such indeterminate nodules. Even if not characterized by a proper texture analysis, this work revealed that this parameter could be an affordable predictor. More recently, *Giovanella et al.* [24] revealed that “Shape_Sphericity” and “GLCM_Autocorrelation” were non redundant predictors for malignancy and a combination of the two features had an AUC of 0.733. Moreover, the authors performed different analysis considering only patients with non-Hürthle cell lesions and all the cohort of the study. In the first group, the two aforementioned RF were independently associated with higher risk of malignancy, with an accuracy for the identification of thyroid cancer of 75%, and an effective predictive model with such parameters was built. When considering all the cohort, the accuracy of the RF was 72% and the association with malignancy and the good performances of the model were confirmed. Lastly, *De Koster et al.* [25] performed a similar study including both Hürtle and non-Hürtle cell nodules and revealed that radiomics did not contribute to the additional differentiation of such nodules, compared to SUV-related parameters.

#### Thyroid cancer

PET/CT is an imaging tool that can be used for the assessment of the biological behavior of thyroid cancer. Particularly, ^18^ F-FDG PET can properly restage aggressive forms of DTC and medullary thyroid cancer (MTC) [35–36]. In this scenario *Lapa et al.* [18] investigated the prognostic value of textural parameters for the assessment of iodine refractory DTC or MTC treated with peptide receptor radionuclide therapy (PRRT). The authors reported a significant correlation for several RF with progression free survival (PFS) and in particular “Grey level non uniformity” was reported as the feature with best performance (AUC 0.930) even if other RF had higher AUCs values. Regarding overall survival (OS), non-significant prognostic RF were reported. Interestingly, in a per-lesion based analysis, only the parameter “Entropy” was able to predict the progression of the lesions (AUC 0.730).

*Nakajo et al.* [20] evaluated the role of radiomics, together with classical SUV-related and volumetric parameters of primary DTC, in the prediction of the risk of recurrence after total thyroidectomy. They reported that patients with high risk of recurrence had higher “IV” and “SVZ” and lower “ZP” values compared to non-high risk subjects and moreover this observation was confirmed in the group of patients with higher metabolic tumor volume (MTV). Furthermore, the same parameters had high AUCs values in the prediction of patients with high risk of recurrence, findings confirmed also in the group of patients with high MTV. Lastly, the authors developed a scoring system for the discrimination between high and non-high risk with a high accuracy.

The pretherapeutic role of radiomics in MTC subjects treated with tyrosine kinase inhibitor was evaluated by *Werner et al.* [21], reporting that a high value of “Complexity” was associated with a reduced OS and that a high value of “Contrast” was correlated with lower PFS; these parameters were also confirmed as affordable prognosticators at multivariate analysis.

### Scintigraphic studies

As previously underlined, ML was also applied to scintigraphic studies in order to assess thyroid pathologies [27–33].

#### Classification of thyroid pathologies

First *Ma et al.* [27] developed a deep convolutional neural network (DCNN) in order to perform thyroid diagnosis based on SPECT images. This method revealed high performances in the differential diagnosis of Grave’s disease, Hashimoto’s thyroiditis and subacute thyroiditis; better performances compared to other method were demonstrated, with higher precision and less classification errors. Similar studies were also proposed by *Qiao et al.* [32] and *Liu et al.* [29] by proposing different DCNN models, revealing high performances for all of them, with AUCs ranging from 0.850 to 0.996. In this setting, an interesting work by *Currie et al.* [30] revealed that ML artificial neural network (ANN) were able to improve the accuracy of the evaluation of thyroid scintigraphy as second readers systems when biochemistry results were available and moreover, deep learning (DL) algorithms were developed to improve the accuracy in the absence of biochemistry results.

A dual center study with similar purpose was performed by *Yang et al.* [33] revealing that a specific DCNN model had the best performances, also confirmed at the external validation. In this setting, the pattern of “heterogeneous uptake” was the most likely to be misclassified and at the external validation this insight was experienced for the “focal increased” uptake pattern.

#### Miscellaneous

An interesting study was performed by *Kavitha et al.* [28] that applied (DL) on post-ablation ^131^I whole body scans in order to assess the presence of metastatic lymph node of DTC. The proposed method revealed the best performances in comparison with the manual detection for both the evaluation of metastatic lymph nodes and the recognition of thyroid remnant tissue. Interestingly, the performances of this method were similar with or without the application of post-processing and had better performances for the recognition of metastatic lymph nodes than physicians at SPECT images.

Lastly, *Guo et al.* [31] evaluated the role of a DCNN model for the classification and diagnosis of residual thyroid tissue at SPECT images, reporting higher performances in comparison to other computer aided diagnosis models with statistically significant differences in particular for sensitivity and accuracy.

## Discussion

Radiomics is defined as the application of different tools for the extraction of quantitative imaging features that reflect the heterogeneity in an image [14, 37], while ML is the scientific discipline that focuses on how computers learn from data and identify some features that are believed to be important for making a final diagnosis [37‒39]. Generally speaking, their role for the assessment of thyroid diseases has been proved in several studies [18–24, 26–34]. Starting with PET/CT imaging, one of the most explored field of application of these technologies was the assessment of TI. In this setting, the studies included in the review [19, 22–23, 26] revealed the selection of some RF as predictor of the final diagnosis of such TIs, with good performances. Moreover, different predictive models with different RF were built and in general high performances for such differential diagnosis were demonstrated. Some attempts to compare different scanner for the assessment of these features and its influence on the final diagnosis were also performed.

Cytologically indeterminate thyroid nodules were also evaluated with radiomics in some studies [24–25, 34], that revealed how some RF were good predictors for the characterization of these nodules even when performing different analysis for Hürthle or non-Hürthle cell lesions. Interestingly, a single study [25] revealed that radiomics did not have a significant role in this field.

In this setting, it is important to mention that in clinical practice there is the option to perform molecular tests, that are useful to define the nature of such nodules. Furthermore, these tests allow the modification of the therapeutic recommendation based on an individualized approach; it has been reported that they could have the ability to rule out the presence of malignancy with great specificity and PPV, avoiding therefore the need to perform unnecessary surgery [39–41].

The role of radiomics analysis on PET/CT was also explored in the field of thyroid carcinomas [18, 20–21] revealing that, in case of iodine refractory DTC or MTC under PRRT therapy, some RF were significantly correlated with the prognosis [18]. Furthermore, some RF were reported as predictive of high risk of recurrence in DTC after total thyroid [20] and some of them were significant prognosticators for OS and PFS in MTC [21].

The role of ML has been also evaluated in scintigraphic imaging [27–33]. In this setting, most of the studies focused on the automatic classification of thyroid disease at scintigraphy applying different DCNN models, reporting in general high performances even in the case of dual center analysis [27, 32–33]. Lastly, good performances of ML models were also obtained when assessing the presence of metastatic lymph nodes and the classification of residual thyroid tissue in DTC patients [28, 31].

Even if our data suggest a role for radiomics and ML in thyroid diseases, these new diagnostic approaches need to be inserted and compared with the current clinical practice. In the case of TI, the high amount of thyroid nodules, the first steps of their evaluation comprehend the measurement of thyroid-stimulating hormone (TSH) levels and US evaluation of the thyroid and the cervical lymph nodes. In this setting, it is known that US is the gold standard for the assessment of thyroid nodules and is therefore mandatory to evaluate the presence of malignant features that will enable the use of fine needle aspiration (FNA), with an accuracy near 95% in determine the presence of malignancy [42–43]. In particular, hypoechoic echogenicity, solid composition, irregular margins, microcalcifications, height greater than width, extrathyroidal extension, disrupted rim calcification, and cervical lymph nodes with suspicious features are elements suspicious for the presence of malignancy. It is worth to underline the fact that our investigation was focused on TI discovered at ^18^ F-FDG PET/CT and in this scenario it has been reported that tracer uptake within a US confirmed thyroid nodule conveys an increased risk of thyroid cancer [44]. In these cases, radiomics could help in the identification of nodules with higher risk.

As mentioned, in the case of indeterminate thyroid nodules (approximately 25% of thyroid FNA samples are classified as Bethesda category 3 or 4), molecular tests can be used to assess the possible presence of malignancy, even if their role in guiding therapeutic decision-making is currently lacking. Moreover, the use of such tests should not be intended to replace other sources of information or clinical judgment [44]. Therefore, even though a benign pattern on molecular testing significantly decreases the risk of malignancy, US surveillance is still required [43]. Again, radiomics and ML could help to underline some features that, together with other information, could be useful to better define the risk of such nodules.

Speaking about patients affected by DTC, total-body scintigraphy with ^131^I, neck US and serum thyrogloblulin measurement are the cornerstones for their clinical follow-up, with high diagnostic accuracy and sensitivity, even in the case of thyroid remnants assessment [44]. However, we should underline that some patients could be classified in the “indeterminate” response group during follow-up and are therefore at risk of relapse [45]. In this setting, radiomics could give some important information able to better classify these patients and setup a specific follow-up.

Lastly, in the case of hyperthyrodidism, a clinical assessment of the patients with subsequent US evaluation, serum assessment of specific antibodies and hormones and scintigraphic evaluation are able to reach a specific diagnosis with proved elevated diagnostic accuracy [2]. The possible role of ML and radiomics in this field seem therefore marginal and wider and stronger studies need to be performed in order to strengthen the value of such diagnostic modalities in daily clinical setting.

Even if, as mentioned, radiomics and ML seems to have a role for the evaluation of thyroid diseases, not all that glitters is gold and many different points and limitations of these technologies and this review need to be underlined. First of all, the problem of repeatability and reproducibility of RF extraction and their subsequent analysis is well known and many efforts in this direction need to be performed in order to clarify this issue. In this setting, it is known that for PET images different scanners used for the acquisition, partial volume effect, reconstruction protocol, tumor segmentation and uptake time are able to affect the textural features extraction and ML [13–14, 26, 37, 38, 46–49]. Another important point that needs to be addressed is the fact that most of the studies included in the review lacked the presence of external validation that is mandatory to strengthen the result obtained in a single center and therefore to establish the clinical significance of radiomics and ML [38, 46–48]. Moreover, it is important to underline that the studies included in the review were performed including different features type and different analysis for their selection, which is an important limit in order to compare their results [37, 49]. Lastly, one of the most important limitations of most of the study evaluated in the review, is the fact that they were performed with limited cohort, which is a big limitation for the evaluation of radiomics potential.

## Conclusion

In conclusion, radiomics and ML seem to have a promising role in the assessment of thyroid diseases. However, many open issues are still present in these fields of research and therefore these results need to be confirmed and standardized in other multicentric settings.
